# Supplementary material for: Maternal carriage of Prevotella during pregnancy associates with protection against food allergy in the offspring
Source: Nat Commun. 2020 Mar 24;11:1452. doi: 10.1038/s41467-020-14552-1 (PMC7093478; doi:10.1038/s41467-020-14552-1)
Supplement: Supplementary file 1 — Supplementary Information [file 41467_2020_14552_MOESM1_ESM.pdf]

SUPPLEMENTARY INFORMATION for Maternal carriage of Prevotella during pregnancy associates with protection against allergic disease in the offspring by Vuillermin et al.

Supplementary Tables

Supplementary Table 1. Baseline characteristics of the study infants

Supplementary Table 2. Types of food sensitization and allergy among offspring

Supplementary Table 3. The output of limma-voom.

Supplementary Table 4. Analysis of the association between maternal carriage of *P. copri* and offspring food allergy mediated by infant 6-month carriage of *P. copri*.

Supplementary Table 5. Analysis of whether there is an association between household size and offspring food allergy mediated by maternal carriage of *P. copri*.

Supplementary Table 6. Primer and Probe Sequences.

Supplementary Figures

Supplementary Figure 1. Hierarchical clustering according to Bray-Curtis dissimilarity based on the relative abundance of OTUs detected maternal fecal samples.

Supplementary Figure 2. Correlation between the relative abundance of OTU697 and OTU41 in maternal fecal samples.

Supplementary Figure 3. Directed acyclic graph representing the posited causal relationship between maternal carriage of *P. copri* and food allergy in the offspring.

Supplementary Figure 4. Alpha diversity according to offspring food allergy status.

Supplementary Figure 5. Bacteroidetes/Firmicutes ratio according to offspring food allergy status.

Supplementary Figure 6. Principal co-ordinate analysis according to offspring food allergy status.

Supplementary Figure 7. The distribution of *P. copri* qPCR expression values.

Supplementary Figure 8. Concentration of short chain fatty acids in fecal samples collected during pregnancy according to offspring food allergy status.

Supplementary Figure 9. Prevalence of maternal carriage of *P. copri* during pregnancy and infant carriage at 1, 6 and 12 months.

Supplementary Figure 10. Maternal dietary intake of fiber in relation to maternal carriage of *P. copri* and offspring allergy.

Supplementary Methods

Supplementary Method 1. Bioinformatics methods

Supplementary Tables

Supplementary Table 1. Baseline characteristics of the study infants

Factor	Inception birth cohort	Random subcohort*	Food allergic
N	1074	256	61
Sex of child			
Female	519 (48.3%)	115 (44.9%)	27 (44.3%)
Male	555 (51.7%)	141 (55.1%)	34 (55.7%)
Twin pairs	10 (0.9%)	2 (0.8%)	1 (1.6%)
Maternal country of birth			
Australia	961 (89.5%)	233 (91.0%)	56 (91.8%)
Other	110 (10.2%)	22 (8.6%)	5 (8.2%)
Unknown	3 (0.3%)	1 (0.4%)	0 (0.0%)
Paternal country of birth			
Australia	915 (85.2%)	220 (85.9%)	49 (80.3%)
Other	108 (10.1%)	23 (9.0%)	8 (13.1%)
Unknown	51 (4.7%)	13 (5.1%)	4 (6.6%)
Maternal age, years, mean (SD)	31.3 (4.8)	32.4 (4.2)	32.2 (4.4)
Paternal age, years, mean (SD)	33.5 (5.9)	34.3 (5.4)	34.4 (5.7)
Maternal education			
Less than year 10	12 (1.1%)	2 (0.8%)	0 (0.0%)
Year 10 or 11 or equivalent	80 (7.4%)	17 (6.6%)	2 (3.3%)
Year 12 or equivalent	162 (15.1%)	36 (14.1%)	12 (19.7%)
Trade/Cert/Dip	266 (24.8%)	62 (24.2%)	12 (19.7%)
Bachelor degree	354 (33.0%)	84 (32.8%)	23 (37.7%)
Postgraduate degree	194 (18.1%)	54 (21.1%)	12 (19.7%)
Unknown	6 (0.6%)	1 (0.4%)	0 (0.0%)
Socioeconomic index for area			
Low	357 (33.2%)	81 (31.6%)	18 (29.5%)
Middle	353 (32.9%)	85 (33.2%)	20 (32.8%)
High	351 (32.7%)	88 (34.4%)	23 (37.7%)
Unknown	13 (1.2%)	2 (0.8%)	0 (0.0%)
Household size during pregnancy			
1	12 (1.1%)	2 (0.8%)	2 (3.3%)
2	416 (38.7%)	82 (32.0%)	22 (36.1%)
3	371 (34.5%)	108 (42.2%)	28 (45.9%)
4	201 (18.7%)	54 (21.1%)	7 (11.5%)
5 or more	69 (6.4%)	10 (3.9%)	2 (3.3%)
Unknown	5 (0.5%)	0 (0.0%)	0 (0.0%)
Maternal cigarette smoking			
Yes	169 (15.7%)	26 (10.2%)	9 (14.8%)
No	892 (83.1%)	230 (89.8%)	52 (85.2%)
Unknown	13 (1.2%)	0 (0.0%)	0 (0.0%)
Pet ownership			
Yes	790 (73.6%)	184 (71.9%)	34 (55.7%)
No	278 (25.9%)	72 (28.1%)	27 (44.3%)
Unknown	6 (0.6%)	0 (0.0%)	0 (0.0%)

First degree family history of eczema	480 (45.8%)	122 (48.4%)	40 (65.6%)
**Factor**	**Inception birth cohort**	**Random subcohort***	**Food allergic**
First degree family history of hayfever	674 (64.4%)	170 (67.7%)	47 (77.0%)
First degree family history of asthma	542 (51.4%)	128 (50.6%)	43 (70.5%)
Delivered in a government hospital			
Yes	780 (72.6%)	168 (65.6%)	41 (67.2%)
No	294 (27.4%)	88 (34.4%)	20 (32.8%)
Delivery via Caesarian section	333 (31.0%)	85 (33.2%)	20 (32.8%)
Gestational age at birth			
32 to <37 weeks	47 (4.4%)	4 (1.6%)	0 (0.0%)
37 to 42 weeks	1027 (95.6%)	252 (98.4%)	61 (100.0%)
Birth weight, grams, mean (SD)	3527 (519)	3564 (498)	3513 (458)
Any BF Completed Weeks, mean (SD)	32.5 (21.1)	33.6 (20.7)	38.2 (18.0)
Age at introduction of solid foods (wks), mean (SD)	19.8 (4.1)	19.6 (3.8)	19.1 (4.4)

* Restricted to infants of mothers with adequate 16S data (≥ 2,500 reads) plus complete/adequate determination of food allergy status at 1 year of age.

Supplementary Table 2. Types of food sensitization and allergy among offspring

Food tested	sensitized ≥2 mm (*n*)	Prevalence sensitized ≥2 mm % 95% CI	Challenge-proven food allergy (*n*)	Food allergy prevalence % 95% CI	Maternal 16S data available (*n*)	*P. copri* in maternal stool (*n*)
All foods	94/806	11.7 (9.5–14.1)	61/788	7.7 (6.0–9.8)	59/61 (97%)	5/59 (8%)
Raw egg	71/820	8.7 (6.8–10.8)	53/811	6.5 (4.9–8.5)	51/53 (96%)	4/51 (8%)
Peanut	28/819	3.4 (2.3–4.9)	13/808	1.6 (0.9–2.7)	12/13 (92%)	1/12 (8%)
Cashew	16/820	2.0 (1.1–3.1)	4/811	0.5 (0.1–1.3)	4/4 (100%)	1/4 (25%)
Cow's milk	9/818	1.1 (0.5–2.1)	4/813	0.5 (0.1–1.3)	4/4 (100%)	0/4 (0%)
Sesame	2/804	0.2 (0.0–0.9)	1/803	0.1 (0.0–0.7)	1/1 (100%)	0/1 (0%)

Supplementary Table 3. The output of limma-voom. This table includes all OTUs identified as differentially abundant in food allergy cases, unadjusted analysis, with q values less than or equal to 0.2, as well as those with the least *q* value greater than 0.2. Ranks are the taxonomic classifications provided by SILVA v123 Nr99. Rows are ordered by (unadjusted) p values (moderated *t*-test in limma).

OTU	Rank7	Rank8	Log fold change	P.Value	Adjusted P.Value
Otu000041	Prevotella_9	uncultured_organism	−0.923	4.13E−06	0.003
Otu000697	Prevotella_9	uncultured_organism	−0.295	4.39E−05	0.015
Otu000026	Methanobrevibacter	NA	−1.126	1.47E−03	0.264
Otu000404	uncultured	uncultured_bacterium	−0.109	2.45E−03	0.264
Otu000069	Ruminococcaceae_UCG−014	NA	−0.767	2.58E−03	0.264
Otu000071	Blautia	NA	0.886	2.58E−03	0.264
Otu000010	Lachnoclostridium	Ruminococcus_gnavus_CC55_001C	1.079	2.68E−03	0.264

Supplementary Table 4. Analysis of the association between maternal carriage of P. copri and offspring food allergy mediated by infant 6-month carriage of P. copri. Adjusted analysis includes maternal consumption of polyunsaturated fats, paternal history of allergy, age at skin prick testing, ethnicity and antenatal dog ownership as for the voom/limma analysis. Effects provided as point estimate of the odds ratio, 95% confidence interval, and *p*-value (Wald test in logistic regression). The proportion mediated is a point estimate only, log(indirect effect)/log(total effect).

	Unadjusted	Adjusted
Direct effect	0.47<br>(0.17, 1.24)<br>0.13	0.49<br>(0.18, 1.36)<br>0.17
Indirect effect	0.90<br>(0.85, 0.96)<br>0.001	0.92<br>(0.77, 1.11)<br>0.374
Total effect	0.42<br>(0.16, 1.14)<br>0.09	0.45<br>(0.16, 1.24)<br>0.12
Proportion mediated	12.2%	10.4%

Supplementary Table 5. Analysis of the association between household size and offspring food allergy mediated by maternal carriage of P. copri. Adjusted analysis includes maternal consumption of polyunsaturated fats, paternal history of allergy, age at skin prick testing, ethnicity and antenatal dog ownership as for the voom/limma analysis. Effects provided as point estimate of the odds ratio, 95% confidence interval, and *p*-value (Wald test in logistic regression). The proportion mediated is a point estimate only, log(indirect effect)/log(total effect), and has wide confidence intervals.

	Unadjusted	Adjusted
Direct effect	0.80<br>(0.55, 1.17)<br>0.25	0.80<br>(0.54, 1.19)<br>0.27
Indirect effect	0.94<br>(0.87, 1.01)<br>0.090	0.94<br>(0.88, 1.01)<br>0.086
Total effect	0.75<br>(0.52, 1.10)<br>0.14	0.75<br>(0.51, 1.12)<br>0.16
Proportion mediated	22.0%	21.5%

Supplementary Table 6. Primer and Probe Sequences

**Primer/Probe Details**		**Sequences**	**Position**	**Amplicon size (bp)**
Universal 16S rRNA gene (MiSeq)	For<br>Rev	5’-GTGCCAGCMGCCGCGGTAA-3’<br>5’-GGACTACHVGGGTWTCTAAT-3’	515-806	292
P. copri, 16S rRNA gene (NR_113411)	For<br>Rev	5’-CGCGAACTGGTTTCCTTGA-3’<br>5’-ACCGCTACACCACGAATTCC-3’	632-687	56
	Taqman probe	FAM-5’-ACGCACAAAGTGGG-3’-MGBNFQ		

SUPPLEMENTARY FIGURES

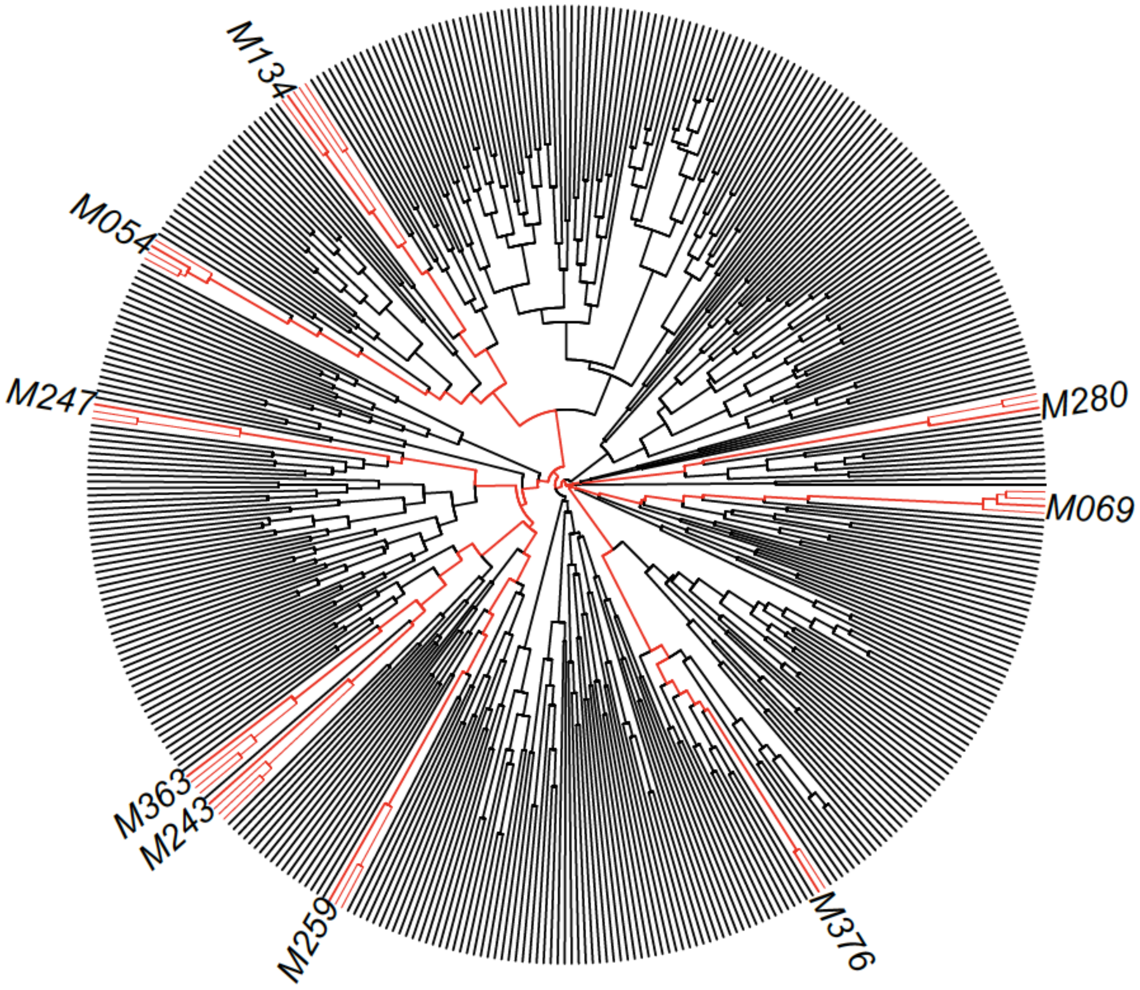

Supplementary Figure 1. Hierarchical clustering according to Bray-Curtis dissimilarity based on the relative abundance of OTUs detected maternal fecal samples. Technical replicates shown in red.

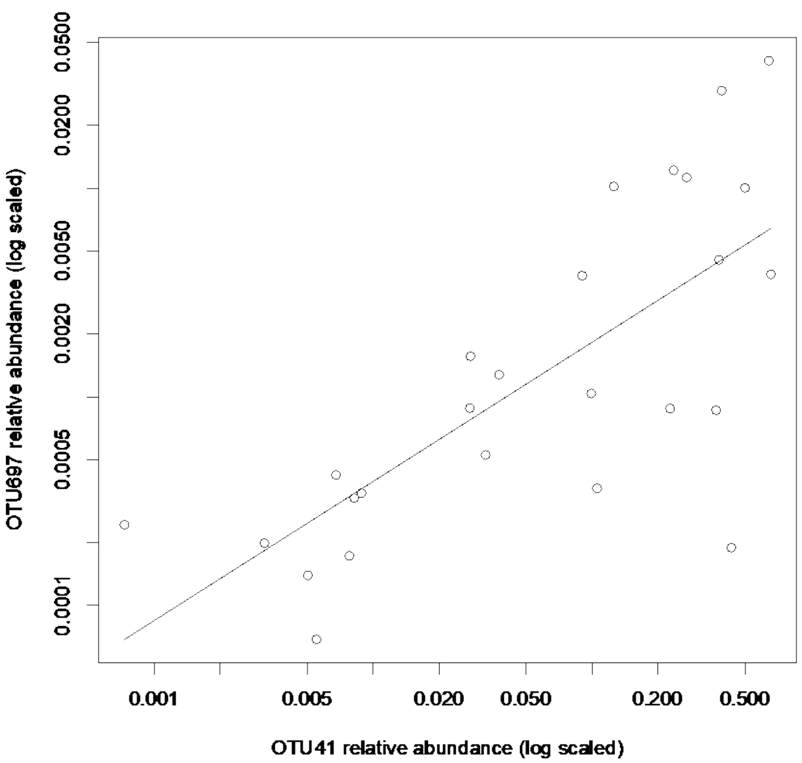

Supplementary Figure 2. Correlation between the relative abundance of OTU697 and OTU41 in maternal fecal samples. Linear regression of log-transformed relative abundance in random subcohort samples where both OTU697 and OTU41 were detected. (Pearson product moment correlation r=0.73; p<0.001 via Wald test in linear regression; n=26). Source data are provided as a Source Data file.

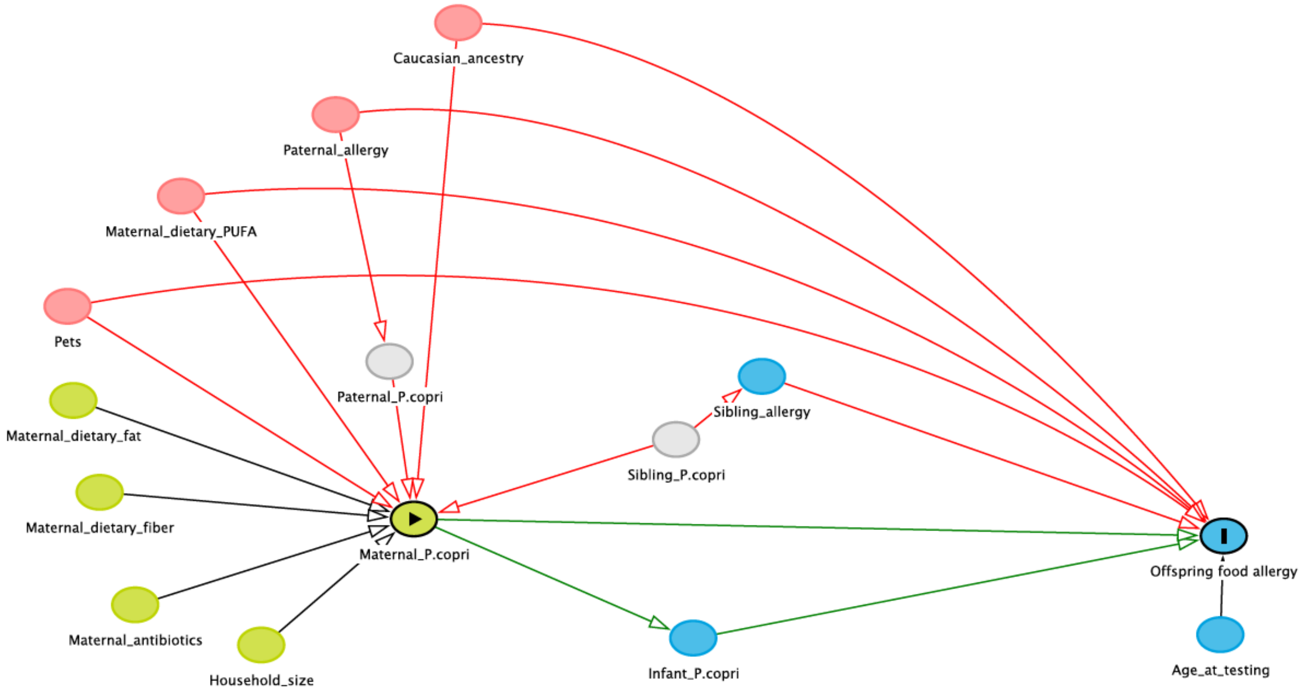

Supplementary Figure 3. Directed acyclic graph representing the posited causal relationship between maternal carriage of *P. copri* and food allergy in the offspring. Pink nodes: potential confounding factors; green nodes: ancestors of the exposure; grey nodes: unobserved factors; blue nodes: ancestors of the outcome.

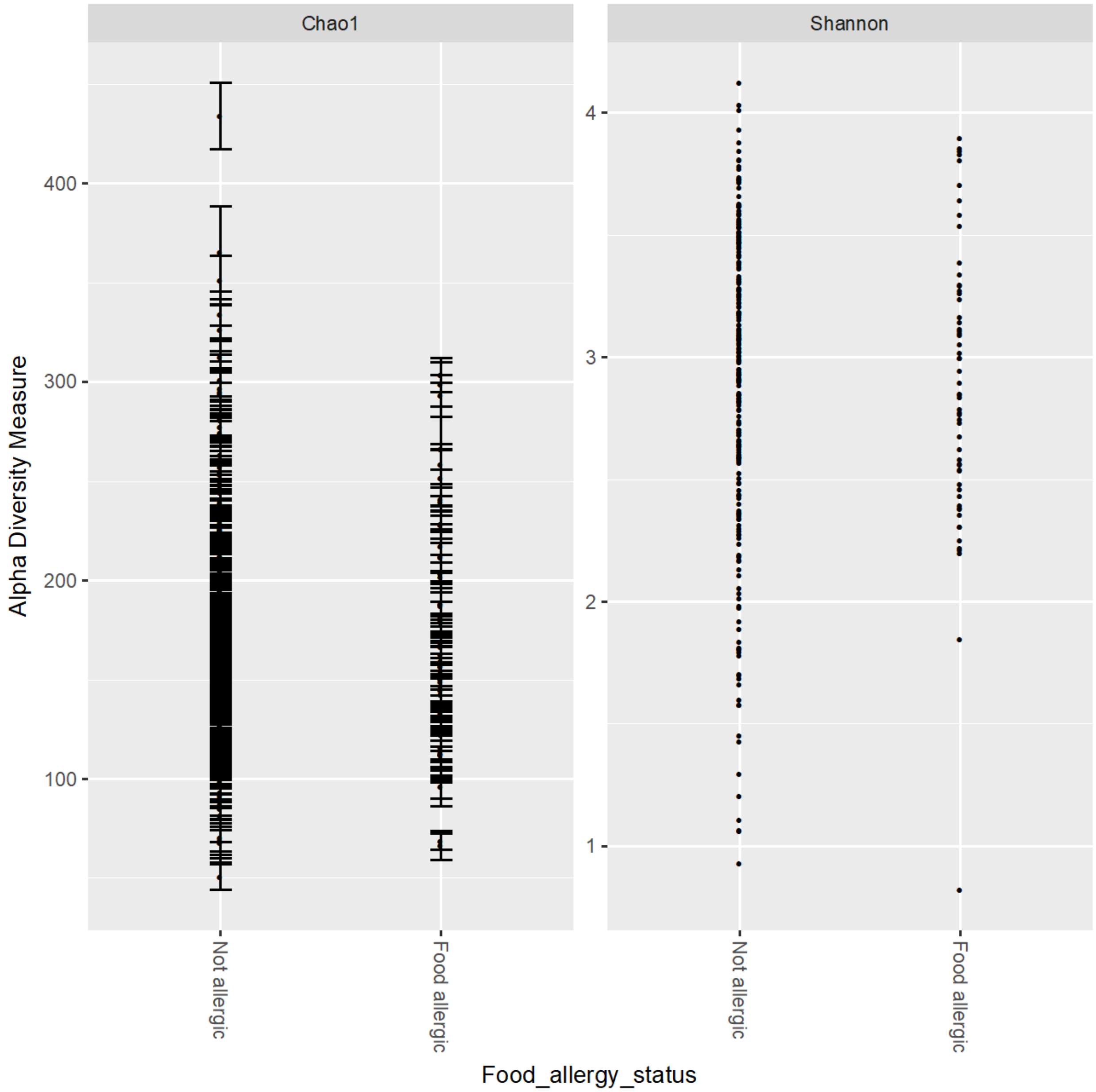

Supplementary Figure 4. Alpha diversity according to offspring food allergy status. Error bars in the Chao1 plot represent ±SE for that measure. The mean Shannon diversities are 2.886 and 2.877 among food allergy controls and cases respectively; 95% CI for the difference is (−0.161, 0.179), p=0.919 (Welch t-test, n=294). Source data are provided as a Source Data file.

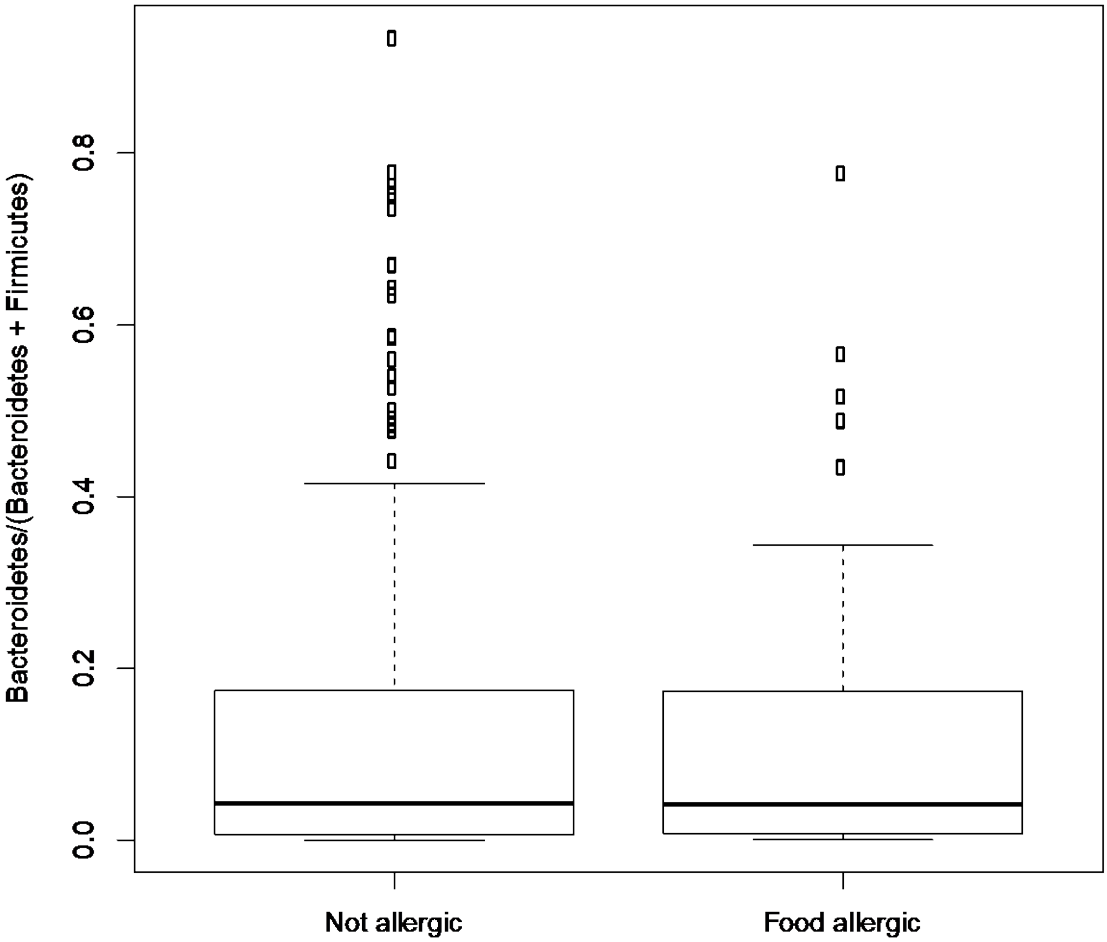

Supplementary Figure 5. Bacteroidetes/Firmicutes ratio according to offspring food allergy status. Tukey boxplots (boxes span quartiles, bold line is the median, upper/lower whiskers cover all datapoints within 1.5 IQR of the upper/lower quartiles respectively, datapoints outside whiskers plotted individually) of the proportion of OTU counts classified as Bacteroidetes as a fraction of the total OTU counts classified as either Bacteroidetes or Firmicutes. Means are 0.130 and 0.118 in food allergy controls and cases respectively; 95%CI for the difference is(−0.038, 0.062), p=0.637 (Welch t-test); p=0.959 via Mann-Whitney test. n=294. Source data are provided as a Source Data file.

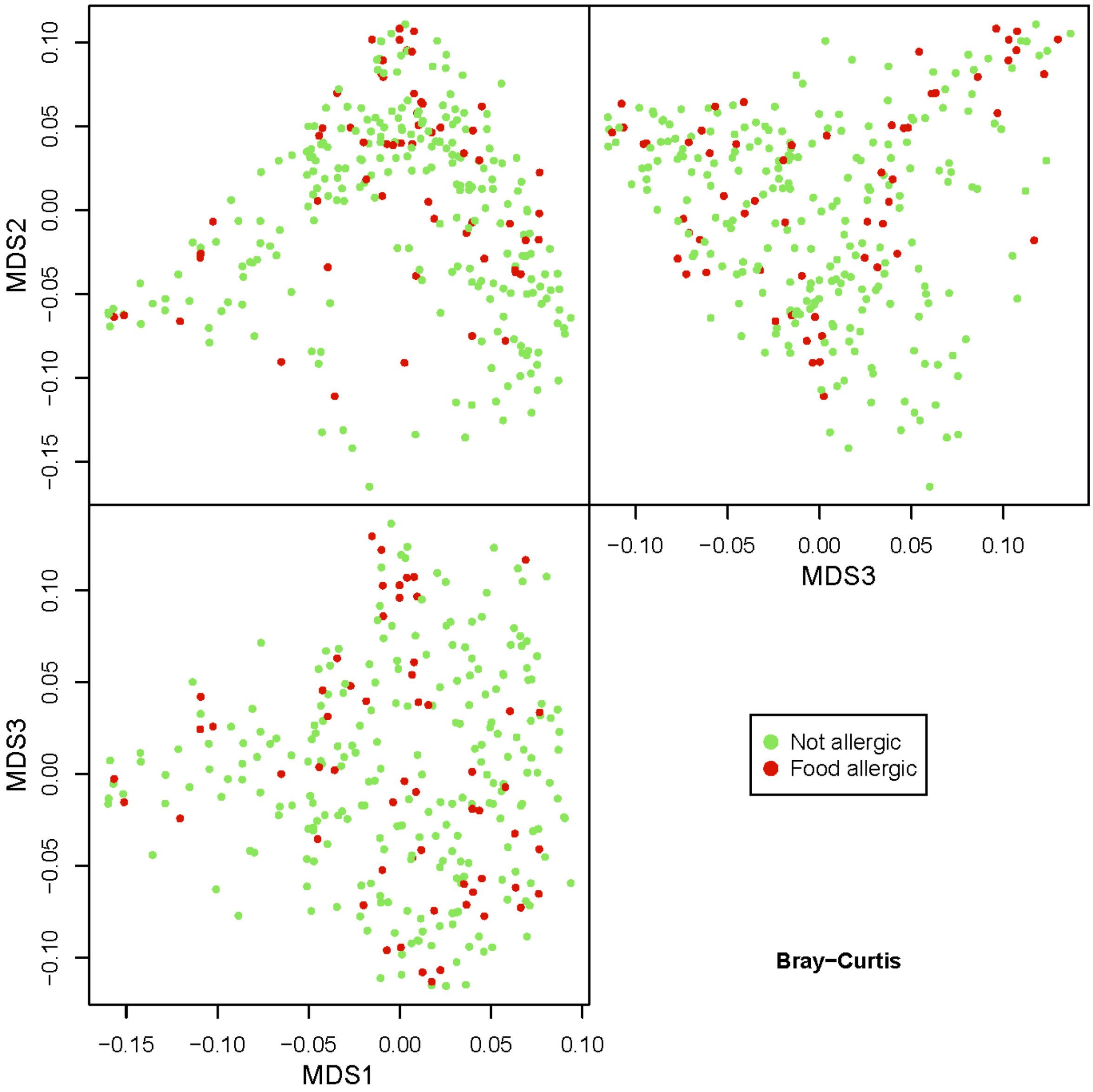

Supplementary Figure 6. Principal co-ordinate analysis according to offspring food allergy status. Plots of the first three principal co-ordinates, utilizing Bray-Curtis dissimilarity. p=0.071 (PERMANOVA, 9999 permutations, n=294). Eigenvalues associated with the first four principal co-ordinates represent 12.4%, 9.0%, 8.5% and 5.0% of the sum of real eigenvalues. Source data are provided as a Source Data file.

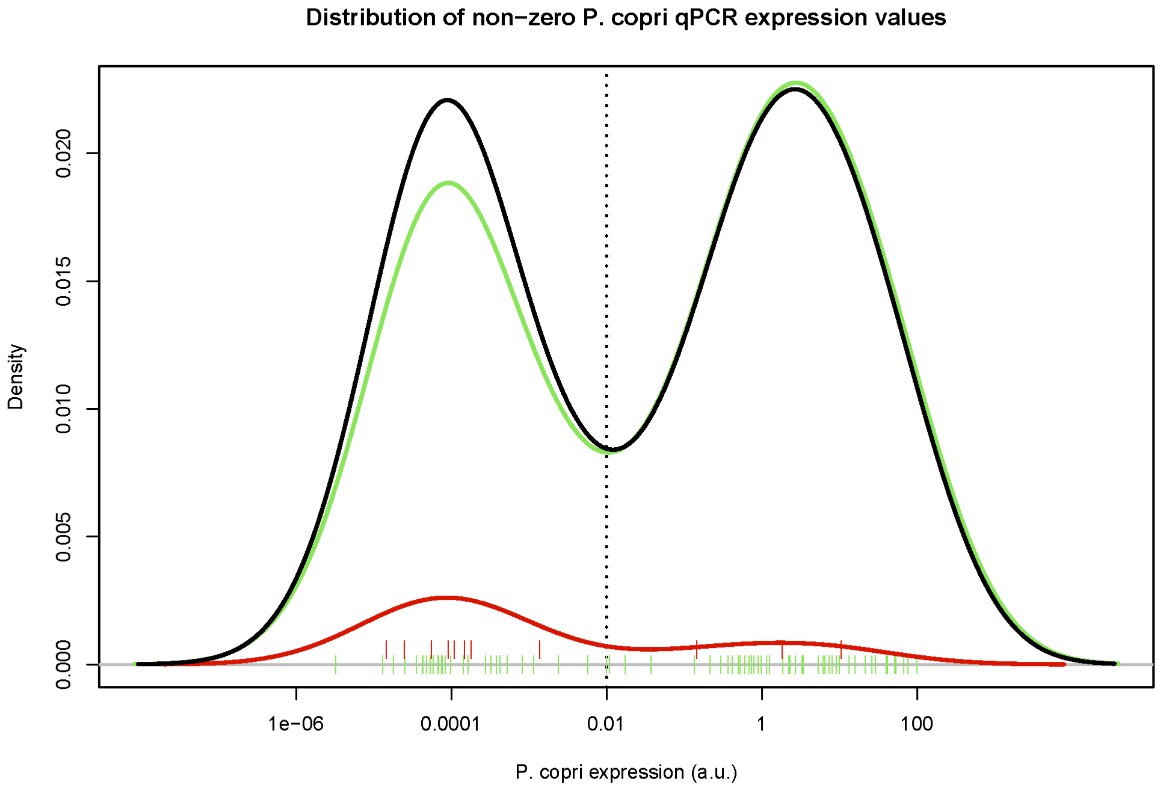

Supplementary Figure 7. The distribution of *P. copri* qPCR expression values. Of 302 samples in the case-subcohort design, 211 had zero *P. copri* expression values (47 of those were food allergic). The remaining 91 are plotted as strokes on the horizontal axis (red: food allergic, raised for clarity; green: not food allergic). The black curve is the gaussian kernel density estimator for all 91 values; the red and green curves are the same for food allergic and not-allergic respectively. The density estimator uses the R default of Silverman's rule-of-thumb to determine the bandwidth parameter. The dotted vertical line shows the 1% cutoff we adopted to define *substantial carriage*. Source data are provided as a Source Data file.

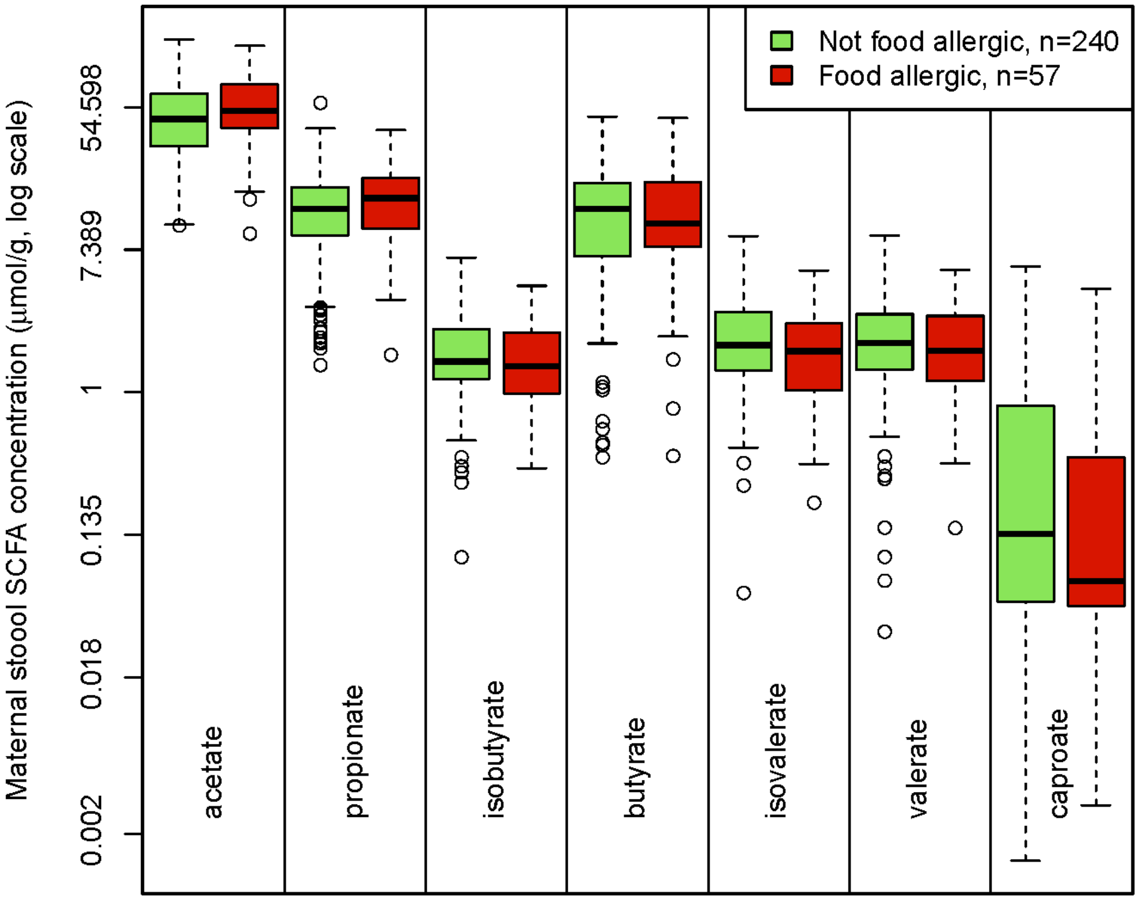

Supplementary Figure 8. Concentration of short chain fatty acids in faecal samples collected during pregnancy among mother according to offspring food allergy status. Tukey boxplots (boxes span quartiles, bold line is the median, upper/lower whiskers cover all datapoints within 1.5 IQR of the upper/lower quartiles respectively, datapoints outside whiskers plotted individually). Source data are provided as a Source Data file.

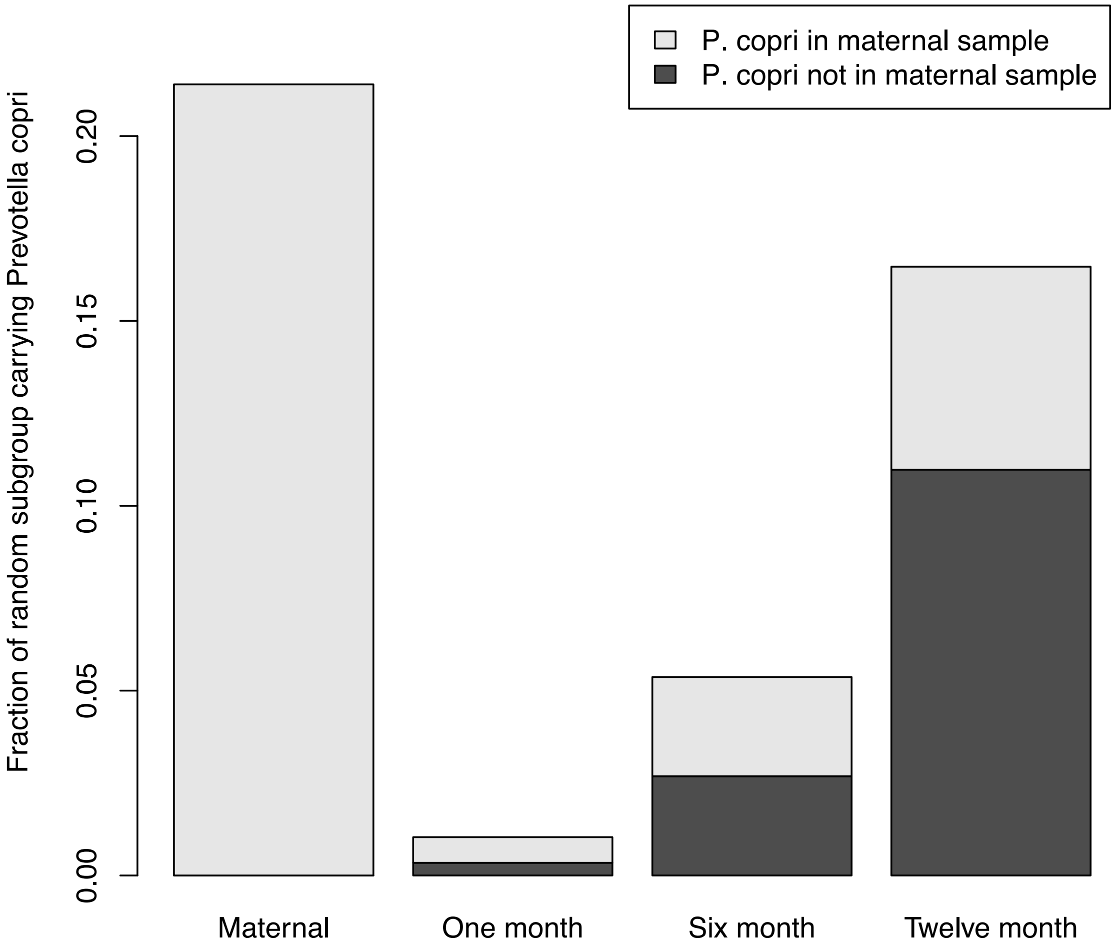

Supplementary Figure 9. Prevalence of maternal carriage of *P. copri* during pregnancy and infant carriage at 1, 6 and 12 months. The correlation between maternal and infant carriage at 1, 6 and 12 months assessed by Kendall's tau was: τ=0.122, 0.195 and 0.191 respectively (p=0.051, 0.002 and 0.005 via the normal approximation to τ, n=257, 261 and 222). Source data are provided as a Source Data file.

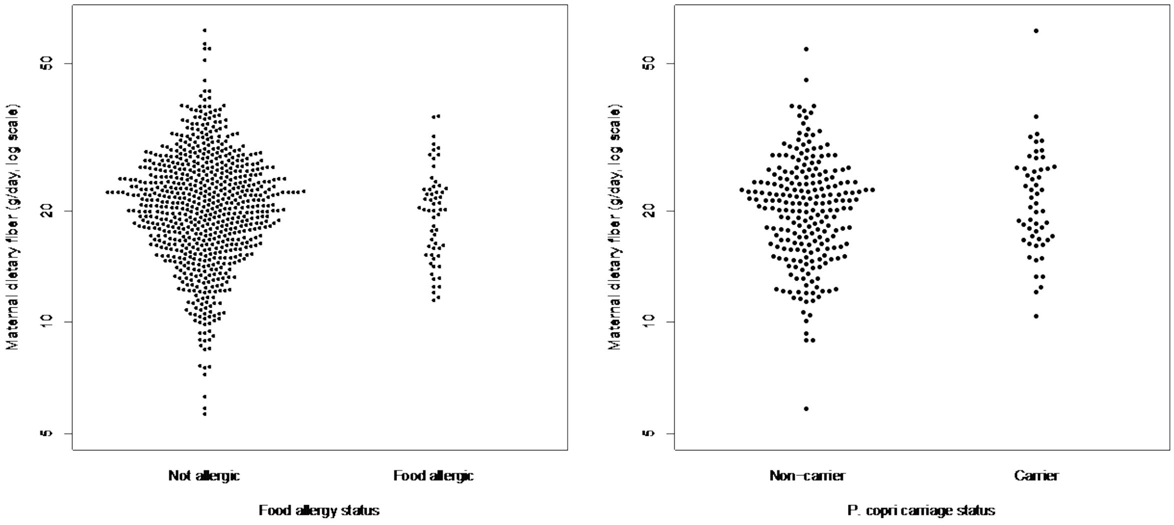

Supplementary Figure 10. Maternal dietary intake of fiber in relation to maternal carriage of *P. copri* and offspring allergy. Odds ratio for food allergy (left panel) 0.97 per 10% increase in dietary fiber (95%CI (0.90, 1.04), *p*=0.37; Wald test in logistic regression; whole cohort with allergy status and maternal fiber consumption available: *n*=772). Odds ratio for *P. copri* carriage (right panel) 1.05 per 10% increase in dietary fiber (95%CI (0.96, 1.15), *p*=0.28; Wald test in logistic regression; food allergy case-random subcohort with maternal fiber consumption available: *n*=284). Source data are provided as a Source Data file.

SUPPLEMENTARY METHOD

Supplementary method 1: Bioinformatics methods

In broad terms, we followed http://joey711.github.io/phyloseq-extensions/edgeR.html to convert our OTU tables to digital gene expression list objects which could then be analyzed as a standard voom/limma workflow, per http://bioconductor.org/packages/release/bioc/vignettes/limma/inst/doc/usersguide.pdf.

The specific procedure included, for each analysis reported:

1. Independent filtering using a variance threshold of 10^{-8}. That is, OTUs whose relative abundance (i.e. raw counts divided by the sample-wise sum of all OTU counts) had variance across samples less than the threshold were excluded from analysis.
2. Relative log expression normalisation(*31*) is performed: scale the counts for each sample by the median across OTUs of the OTU count divided by the geometric mean of that OTU's count across all samples.
3. For each OTU a linear model is fitted using the covariates (i.e. the main predictor of interest as well as those we wished to adjust for) as predictors and the normalized pseudocounts as responses.

The filtered and normalized OTU table is then passed to limma and processed with voom(*35*)

4. Lowess smoothing is applied to the square-root-standard-deviation for each OTU as a function of its mean response.
5. For each OTU in each sample, a precision weight is calculated as the inverse of the variance described by the lowess curve as a function of the predicted response.

These weights are then incorporated into the standard limma pipeline.

6. Linear models are fitted for each OTU, this time incorporating the precision weights.

7. Details of the fit for the covariate of interest are extracted.
8. Empirical Bayes moderation(*36*) is performed yielding moderated *t* statistics for the fits.
9. *p* values corresponding to the moderated *t* statistics are computed.
10. The *p* values are adjusted according to the Benjamini-Hochberg procedure.
