# Supplementary material for: Maternal carriage of Prevotella during pregnancy associates with protection against food allergy in the offspring
Source: Nat Commun. 2020 Mar 24;11:1452. doi: 10.1038/s41467-020-14552-1 (PMC7093478; doi:10.1038/s41467-020-14552-1)
Supplement: Supplementary file 3 — Reporting Summary [file 41467_2020_14552_MOESM3_ESM.pdf]

nature research

Corresponding author(s): Peter J Vuillermin

Last updated by author(s): Nov 20, 2019

Reporting Summary

Nature Research wishes to improve the reproducibility of the work that we publish. This form provides structure for consistency and transparency in reporting. For further information on Nature Research policies, see Authors & Referees and the Editorial Policy Checklist .

Statistics

For all statistical analyses, confirm that the following items are present in the figure legend, table legend, main text, or Methods section.

n/a	Confirmed	
☐	☒	The exact sample size (*n*) for each experimental group/condition, given as a discrete number and unit of measurement
☐	☒	A statement on whether measurements were taken from distinct samples or whether the same sample was measured repeatedly
☐	☒	The statistical test(s) used AND whether they are one- or two-sided<br>*Only common tests should be described solely by name; describe more complex techniques in the Methods section.*
☐	☒	A description of all covariates tested
☐	☒	A description of any assumptions or corrections, such as tests of normality and adjustment for multiple comparisons
☐	☒	A full description of the statistical parameters including central tendency (e.g. means) or other basic estimates (e.g. regression coefficient) AND variation (e.g. standard deviation) or associated estimates of uncertainty (e.g. confidence intervals)
☐	☒	For null hypothesis testing, the test statistic (e.g. *F*, *t*, *r*) with confidence intervals, effect sizes, degrees of freedom and *P* value noted<br>*Give P values as exact values whenever suitable.*
☒	☐	For Bayesian analysis, information on the choice of priors and Markov chain Monte Carlo settings
☒	☐	For hierarchical and complex designs, identification of the appropriate level for tests and full reporting of outcomes
☐	☒	Estimates of effect sizes (e.g. Cohen's *d*, Pearson's *r*), indicating how they were calculated

Our web collection on statistics for biologists contains articles on many of the points above.

Software and code

Policy information about availability of computer code

Data collection	No software was used to collect the data in this study
Data analysis	usearch v8.1.1861_i86linux64<br>mothur v.1.36.1<br>Stata v 15.1<br>R v 3.6.1

For manuscripts utilizing custom algorithms or software that are central to the research but not yet described in published literature, software must be made available to editors/reviewers. We strongly encourage code deposition in a community repository (e.g. GitHub). See the Nature Research guidelines for submitting code & software for further information.

Data

Policy information about availability of data

All manuscripts must include a data availability statement. This statement should provide the following information, where applicable:

- Accession codes, unique identifiers, or web links for publicly available datasets
- A list of figures that have associated raw data
- A description of any restrictions on data availability

Microbiota sequencing reads have been submitted to the Sequence Read Archive under accession number PRJNA576314 [https://www.ncbi.nlm.nih.gov/sra/?term=PRJNA576314]. Access to BIS data including all data used in this paper can be requested through the BIS Steering Committee by contacting the corresponding author. Requests to access cohort data are considered on scientific and ethical grounds and, if approved, provided under collaborative research agreements. Deidentified cohort data can be provided in Stata or CSV format. Additional project information, including cohort data description and access procedure, is available at the project's website https://www.barwoninfantstudy.org.au. Source data underlying Figures 2–5 and Supplementary Figures 2 and 4–10 have been provided as a Source Data file.

Field-specific reporting

Please select the one below that is the best fit for your research. If you are not sure, read the appropriate sections before making your selection.

- [x] Life sciences
- [ ] Behavioural & social sciences
- [ ] Ecological, evolutionary & environmental sciences

For a reference copy of the document with all sections, see nature.com/documents/nr-reporting-summary-flat.pdf

Life sciences study design

All studies must disclose on these points even when the disclosure is negative.

Sample size	Sample size was determined by (i) the larger Barwon Infant Study (BIS) cohort size (1064 mothers/1074 children), (ii) the number of those children with food-challenge proven food allergy at 12 months, (iii) the principal BIS subcohort (321 mothers/324 children randomly selected from those participating in the 12 month review), (iv) availability of a maternal stool sample. The case group of food allergic children was maximal within the cohort. Families within the subcohort with a non-food allergic child were a more than sufficient control group at 5 times the size of the case group.
Data exclusions	Data on an individual were excluded if (i) fewer than 2500 sequencing reads were obtained from the individual (excluding clearly low outliers based on examination of the sequencing data and without reference to case status), (ii) determination of food allergy status was inconclusive (pre-established).
Replication	Some level of replication was provided by the real-time PCR portion of the study. A full replication was infeasible as it would involve recruiting a large number of participants and skin prick testing / food challenge on offspring.
Randomization	Not relevant in this observational study.
Blinding	Blinding was not possible due to the relatively small number of individuals in the case group.

Reporting for specific materials, systems and methods

We require information from authors about some types of materials, experimental systems and methods used in many studies. Here, indicate whether each material, system or method listed is relevant to your study. If you are not sure if a list item applies to your research, read the appropriate section before selecting a response.

Materials & experimental systems

n/a	Involved in the study	
☒	☐	Antibodies
☒	☐	Eukaryotic cell lines
☒	☐	Palaeontology
☒	☐	Animals and other organisms
☐	☒	Human research participants
☒	☐	Clinical data

Methods

n/a	Involved in the study	
☒	☐	ChIP-seq
☒	☐	Flow cytometry
☒	☐	MRI-based neuroimaging

Human research participants

Policy information about studies involving human research participants

Population characteristics	Participants were recruited as pregnant women at least 18 years old using an unselected sampling frame.
Recruitment	Pregnant women attending an antenatal clinic at either of two local hospitals were invited to participate in BIS. Participants were more likely to report a first-degree relative with asthma or eczema; we expect this would bias our findings towards the null.
Ethics oversight	The study was approved by the ethics committee at Barwon Health, Geelong, Victoria, Australia.

Note that full information on the approval of the study protocol must also be provided in the manuscript.
